# Assessing the family dynamics of childhood maltreatment history with the Childhood Attachment and Relational Trauma Screen (CARTS)

**DOI:** 10.3402/ejpt.v6.27792

**Published:** 2015-08-03

**Authors:** Paul Frewen, Matthew Brown, Jonathan DePierro, Wendy D'Andrea, Allan Schore

**Affiliations:** 1 Departments of Psychiatry, Psychology, and Graduate Program in Neuroscience, Western University Canada, London, Ontario, Canada; 2 Department of Psychology, Western University, London, Ontario, Canada; 3 Department of Psychology, The New School for Social Research, New York, NY, USA; 4 Department of Psychiatry and Biobehavioral Sciences, David Geffen School of Medicine, University of California, Los Angeles, CA, USA

**Keywords:** Childhood abuse and neglect, childhood maltreatment, childhood trauma, attachment, posttraumatic stress disorder

## Abstract

**Background:**

Existing survey measures of childhood trauma history generally fail to take into account the relational-socioecological environment in which childhood maltreatment occurs. Variables such as the relationship between the perpetrator and the victim, the emotional availability of caregivers, witnessing the abuse of others, and the respondent's own thoughts, feelings, and actions in response to maltreatment are rarely assessed by current measures.

**Methods:**

To address these concerns, the current study further investigated the family dynamics of childhood maltreatment using the Childhood Attachment and Relational Trauma Screen (CARTS) in 1,782 persons assessed online.

**Results:**

Paired differences in means between item-rated descriptiveness of self, mothers, and fathers suggested that respondents’ relationship with their biological fathers was less positive and secure than their relationship with their biological mothers, and that biological fathers were more often the perpetrator of emotional, physical, and sexual abuse than biological mothers. However, results further suggested that ratings between self, mothers, and fathers were positively correlated such that, for example, reports of a mother's or a respondent's own abusive behavior were more likely in the presence of reports of a father's abusive behavior. In addition, analyses evaluating witnessing violence demonstrated that fathers were rated as more often violent toward mothers than the reverse, although intimate partner violence was also frequently bidirectional. Analyses of sibling ratings further demonstrated that older brothers were either as or more frequently abusive when compared with parents. Finally, results suggested that childhood emotional, physical, and sexual abuse were much more often perpetrated by family members than extra-familial and non-family members.

**Conclusions:**

In so far as these findings are consistent with the prior childhood trauma and attachment literature, the current study further supports the utility of the CARTS as a means of assessing the family dynamics of childhood attachment and maltreatment within a relational-socioecological framework.

Secure attachments and emotional bonds with caregivers during childhood are thought to be protective against the development of mental health problems later in adulthood (Schore, 1994, 2001, 2003a, 2003b, 2012, 2014). Besides investigations of familial abuse and neglect perpetrated by parents, research and clinical attention toward intersibling violence is also increasing due to greater recognition of its prevalence and sequelae (Duncan, 1999; Skinner & Kowalski, 2013; Tippett & Wolke, 2014; Turner, Finkelhor, & Ormrod, 2010). For example, Button and Gealt (2010) found that physical violence at the hands of siblings in childhood had double the prevalence of physical violence perpetrated by parents, and increased the odds of later delinquency, substance abuse, and aggression. Bowes et al. (2014) found that sibling violence prospectively predicted and increased the odds of future depression (OR=2.56), anxiety (OR=1.83), and self-harm (OR=2.56), and these effects were only mildly attenuated by a range of confounding variables including maltreatment by an adult, witnessing domestic abuse, peer victimization, and pre-existing emotional and behavioral problems. Such findings suggest that the effects of sibling violence are both significant and unique (see also Tucker, Finkelhor, Turner, & Shattuck, 2013). The literature on sibling conflict also highlights the necessity of assessing multiple family members for the same type of abuse or ill-treatment, recognizing that there is likely an interaction between interparental conflict and intersibling conflict, and that their co-occurrence results in a generally more hostile and insecure familial environment (Ingoldsby, Shaw, & Garcia, 2001; Tucker et al., 2013; Volling & Belsky, 1992). For example, Hoffman and Edwards (2004) argue that sibling conflict is interdependent with negative interaction and behaviors occurring among all family members. Hoffman and Edwards’ framework highlights the assessment of the socioecological environment in which sibling conflict occurs, taking into account the characteristics of the parents’ relationship, the parent-child relationship, the siblings’ relationship, and the individual thoughts and attitudes of the respondent (Hoffman, Kiecolt, & Edwards, 2005).

A growing literature suggests that witnessing violence can also have a significant impact on a wide range of adverse psychological outcomes (Evans, Davies, & DiLillio, 2008; Kitzmann, Gaylord, Holt, & Kenny, 2003; Teicher & Vitaliano, 2011). For example, children who witness domestic violence are more likely to come from homes where there are low levels of warmth between family members, poorer relationships between parents, and poorer relationships between parents and children (Hamby, Finkelhor, Turner, & Ormrod, 2010; Lepistö, Luukkaala, & Paavilainen, 2011). Child witnesses to domestic violence are also at increased risk for various trauma-related disorders, such as posttraumatic stress disorder, depression, and substance-use disorders (Kilpatrick & Williams, 1997; Spilsbury et al., 2007; Teicher, Samson, & Polcari, 2006). In addition, difficulties in broad internalizing and externalizing domains are generally found (e.g., emotion regulation difficulties, conduct problems; Kennedy, Bybee, Sullivan, & Greeson, 2009; Mrug & Windle, 2010; Russell, Springer, & Greenfield, 2010; Spilsbury et al., 2007). In fact, Teicher and Vitaliano (2011) found that witnessed parental violence toward siblings had greater adverse effects on psychological well-being than parental violence directed toward oneself.

The Childhood Attachment and Relational Trauma Screen (CARTS; Frewen et al., 2013) is a recently developed retrospective measure of the relational matrix and family dynamics within which incidences of childhood maltreatment often occur (e.g., the presence vs. absence of a caretaker, the quality of sibling relationships). Moreover, the CARTS assesses not only maltreatment occurrences but further the positivity, warmth, and support shared between family relationships, including in the form of the emotional availability of caregivers to their children and the proximity seeking of children to their caregivers during times of distress. In addition, the CARTS specifically assesses maltreatment-related thoughts, feelings, and behaviors, as these experiences predict additional variance in psychological outcomes over level of trauma exposure alone (Martin, Cromer, DePrince, & Freyd, 2011).

More specifically, the CARTS uses a relationally contextualized survey methodology that asks what items apply as descriptions of the respondents’ family members. The CARTS also asks the respondent to indicate whether survey items apply as a description of him or herself. For example, an item such as “I was physically abused” would instead be phrased “This person was physically abusive,” and respondents’ would simultaneously assess item applicability as a description of multiple family members (e.g., mother, father, siblings, as well as in reference to the participant him or herself). Specificity regarding abuser characteristics is particularly relevant given that rates of abuse are known to differ by type of family member. For example, research suggests that mothers are more often a sole perpetrator of emotional abuse and neglect when compared with fathers, whereas the reverse is true in the case of childhood sexual abuse (e.g., Finkelhor, Vanderminden, Turner, Hamby, & Shattuck, 2014).

However, only a single report has so far investigated the utility of the CARTS in exploring the family dynamics of childhood maltreatment (Frewen et al., 2013). Moreover, that study was limited by the use of relatively small samples, and only investigated occurrences of childhood trauma and neglect perpetrated by parents. Therefore, the prior study failed to acknowledge the role of intersibling violence, witnessing violence, as well as abuse and neglect perpetrated by extended family or persons outside the family. The primary purpose of the current research effort was, therefore, to further evaluate the CARTS as a methodology for investigating the family dynamics of childhood trauma in a large general population sample by extending analysis to ratings referring to siblings, extended family, and persons external to the family. We investigated the convergent validity of the CARTS in relation to the Childhood Trauma Questionnaire (CTQ) (Bernstein & Fink, 1998) and the Juvenile Victimization Questionnaire (JVQ) (Adult Self-Report Version; Hamby, Finkelhor, Ormrod, & Turner, 2005), and evaluated the concurrent predictive validity of the CARTS in relation to self-reported DSM-5 PTSD symptoms by administering the PTSD Checklist (PCL-5; Weathers et al., 2013). More substantively, we assessed the family dynamics of childhood maltreatment by comparing CARTS item endorsements as referring to self, parents, and siblings. Moreover, we revised the CARTS response format to allow ratings to be collected as referring to persons outside those of the respondents’ family and compared the prevalence of childhood emotional, physical, and sexual abuse perpetrated by “nuclear” familial vs. extra-familial vs. non-familial relationship status with the prediction that familial perpetrators would be identified more frequently. We reasoned that, to the extent that obtained results replicate trends that can be predicted from the prior research literature as such, the present analyses would further support the utility of the CARTS as a means of investigating the socioecology of childhood trauma occurrences within a relationally contextualized assessment framework.

## Methods

### Participants

A total of 2,728 participants visited an external SSL encrypted website as recruited from Amazon's Mechanical Turk (MTurk) Webservice in order to complete a survey battery assessing trauma exposure and trauma-related outcomes, included within the CARTS. The MTurk online participation portal represents a diverse, international workforce of more than a half-million persons (Paolacci & Chandler, 2014; Shapiro et al., 2013). Research suggests that MTurk participants do not differ significantly from general populations excepting that they are somewhat younger (on average 30 years old), more educated, and more frequently unemployed (Goodman, Cryder, & Cheema, 2013; Paolacci & Chandler, 2014; Paolacci, Chandler, & Ipeirotis, 2010). Shapiro et al. (2013) surveyed experiences of trauma exposure, depression, and anxiety disorders in MTurk participants and found that endorsement rates are generally representative of population rates, with certain elevations. They also found that participants reported greater comfort disclosing clinical information including trauma history in an online format than they would have in an in-person interview.

Following Frewen et al. (2013), the sole study inclusion criterion was that participants enter data on both their biological parents. Of the 2,728 participants recruited, 2,258 completed the CARTS in full (82.6% completion rate). However, of these, 476 participants (17.4%) did not enter data on both their biological mother and biological father and so were excluded from further analysis. This procedure resulted in a final sample of 1,782 participants (see Table 1 for description of sample). The only difference between CARTS completers and dropouts on measured demographic variables was that completers were more likely to report a history of psychiatric illness (19.3% vs. 14.1%, *p*=.05). The completer sample of 1,782 was divided into three different subsamples (*n*=726; *n*=457; *n*=599) who, while all completing the CARTS, were administered different additional measures of childhood maltreatment to assess convergent validity (CTQ, JVQ, or CTQ-S; see further description below). Of the 1,782 participants who included demographic information on both biological parents, 1,364 (76.5%) also included data referring to at least one sibling. Across study subsamples, participants reported on average 1.14 (*SD*=1.03) brothers and 0.89 (*SD*=0.95) sisters.

**Table 1:** Demographic characteristics of full MTurk sample (*n*=1,782)

	MTurk participants
Variable	*N* (%) or *M* (SD)
Age	33.96 (11.54)
Sex	
Female	1,220 (67.9)
Male	554 (31.4)
Declined	8 (0.7)
Race	
Caucasian	1,381 (77.5)
Mixed race selected	133 (7.5)
Any other specific race selected (e.g., Northeast Asian)	96 (5.4)
Other race selected	111 (6.2)
Declined	61 (3.4)
Marital status	
Single	787 (44.2)
Married or common-law	732 (41.1)
Divorced or separated	182 (10.2)
Widowed	14 (0.8)
Other	48 (2.7)
Declined	19 (1.1)
Education level (highest attained):	
Completed post-secondary	971 (54.5)
Completed high school	154 (8.6)
Partially completed post-secondary	632 (35.5)
Did not complete high school	14 (0.8)
Declined	11 (0.6)
Employment	
Full- or part-time	949 (53.3)
Unemployed	279 (15.7)
Student	221 (12.4)
Self-employed	187 (10.5)
Unable to work	84 (4.7)
Other	47 (2.6)
Declined	15 (0.8)
Diagnosed psychiatric history	
No history of psychiatric diagnosis	927 (52.0)
Current psychological diagnosis	453 (25.4)
Past psychiatric diagnosis	344 (19.3)
Declined	58 (3.3)
Family description	
Includes one brother	640 (46.9)
Includes one sister	555 (40.7)
Includes more than one brother	368 (27.0)
Includes more than one sister	272 (19.9)

### Questionnaires administered

#### Childhood Attachment and Relational Trauma Screen

The CARTS (Frewen et al., 2013) is a computer-based self-report measure designed to assess overt instances of childhood maltreatment (i.e., physical and emotional abuse of self or other family members, sexual abuse toward the respondent, and “bad things” possibly occurring), as well as the general warmth, security, and supportiveness of individuals within the respondents’ family and external environment. Item content also permits for certain items to be applicable to the respondent him/herself. It is worth emphasizing that the CARTS assessment procedure not only includes verbal stimuli (survey items) but also a non-verbal (visual) modality (participants’ family members are identified with stick figures) which may activate processing within the right hemisphere, thought to be dominant for attachment representations, whether secure or traumatic in nature (e.g., Schore, 2014).

Specific items from the original CARTS 56-item list can be seen in Frewen et al. (2013). These items were collapsed into 13 subscales that evidenced good internal consistency reliability, though with some exceptions depending upon the type of family relationship rated, as reported by Frewen et al. (2013). Specifically, milder physical abuses involving being “slapped, smacked, or hit” by a family member did not correlate as strongly with more severe abuses involving being “punched or kicked” by the same family member as one would prefer as grounds for substantiating the simple summation of responses to the two questions as a “Physical Abuse” subscale. This was similarly the case in reference to a “Negative Affect” subscale comprised of responses characterizing a person as frequently “sad,” “mad,” and “scared,” particularly in reference to mothers. Finally, the internal consistency of mother ratings was lower than that observed for father ratings for items describing “bad things happening” to the respondent, due to very few respondents in the previous study indicating that their mothers “made [them] do bad things that [they were] not supposed to tell other people about.” However, for the sake of parsimony (e.g., to obviate the need to analyze two levels of severity of physical abuse, or tendencies toward three distinct negative emotions), as well as to afford direct comparison with previous samples, the current study followed the simplified subscale scoring recommended by Frewen et al. (2013). The prior study also demonstrated the convergent validity of the CARTS through positive correlations with the CTQ (Bernstein & Fink, 1998).

In addition to the 56 items used in the previous study, the current study included eight additional items intended to assess the experience of witnessing domestic violence (see Appendix). Toward this end, highly face valid items were constructed in order to assess the experience of witnessing violence by the respondents’ mother, father, brother(s), and sister(s) (e.g., “I witnessed (watched or heard) this person being threatened or assaulted by My Mother”), and the witnessing of violence directed toward the respondents’ mother, father, brother(s), or sister(s) (i.e., each person[s] separately) (e.g., “This person threatened or assaulted My Mother”). Note that the construction of such items is consistent with a relational perspective on childhood maltreatment, in that items not only assess that the respondent witnessed violence, but further request information regarding who was violent, and toward whom.

Administration of the CARTS was fully automated by computer via the internet and identical to that described in Frewen et al. (2013) with the exception of two important caveats. First, the upper limit for ratings of family members was increased from 11 as utilized in prior research to 20 in the present study to accommodate participants who may wish to make ratings for a larger number of family members or include a greater number of non-family members (e.g., friends) within their family listing. This is relevant given that the CARTS specifically instructs participants that they may include whomever they wish within the composition of their family. Further along these lines, a second revision involved giving participants the opportunity to indicate that a statement applied to someone not previously included in their family listing during survey completion by clicking a button labelled “Include someone not already listed”; as such, critically, the revised methodology made more possible the flexible inclusion of ratings referring to non-family members.

### Measures of convergent validity

#### Childhood Trauma Questionnaire

The CTQ (Bernstein & Fink, 1998) is a 28-item self-report instrument that measures experiences of emotional (*α*=.91), physical (*α*=.85), and sexual abuse (*α*=.96), as well as experiences of emotional (*α*=.91) and physical neglect (*α*=.81). Responses were made on a five-point Likert scale ranging from 0 to 5 (Never True to Very Often True), indicating severity of experiences.

#### Childhood Trauma Questionnaire - Screen

This included only four items from the CTQ, two of which were previously validated (Thombs, Bernstein, Ziegelstein, Bennett, & Walker, 2007) for screening history of physical abuse (i.e., “People in my family hit me so hard that it left me with bruises or marks”) and sexual abuse (i.e., “Someone tried to touch me in a sexual way, or tried to make me touch them”). Following Frewen et al. (2013), we also utilized a face valid screening item for emotional abuse history (i.e., “I believe that I was emotionally abused”), and presented but did not analyze a filler item assessing general satisfaction with familial upbringing (“i.e., My family was a source of strength and support”). Responses were made on the same five-point scale as used for the CTQ.

#### Juvenile Victimization Questionnaire - Adult Self-Report

The JVQ-AR (Hamby, Finkelhor, Ormrod, & Turner, 2005) is a 34-item measure designed to assess a broad range of childhood victimization experiences including childhood maltreatment, experiences of criminal victimization, sexual assault, bullying, and witnessing violence. Responses were made on a six-point Likert scale anchored from 0 (No) to 5 (five times or more). Previous research has delineated responses to the JVQ-AR into five subscales (i.e., Child Maltreatment, Conventional Crime, Peer and Sibling Victimization, Sexual Victimization, and Witnessing Violence). For the purposes of this study we examined only the Child Maltreatment scale (JVQ-M, *α*=.74).

### Measures of concurrent predictive validity

#### PTSD Checklist-5

The PCL-5 (Weathers et al., 2013) contains 20 items which capture the DSM-5 diagnostic criteria of PTSD. Responses are made on a scale from 0 (Not at all) to 4 (Extremely) based on how much a respondent was bothered by a given symptom over the past week. Internal consistency for the PCL-5 total score in the current sample was *α*=.96.

### Statistical analysis

In order to replicate and extend previous research with the CARTS (Frewen et al., 2013), we first examined four response classes that were available for all participants as a study inclusion criterion, specifically, items considered true for: 1) a respondent him/herself; 2) a respondent's biological mother; 3) a respondent's biological father; and 4) items considered not applicable to any member of the respondent's family (i.e., for which participants clicked the “Not Applicable” box). It is important to note that the “Not Applicable” selection refers not only to the “non-applicability” of the item to Self, Mother, and Father, but rather to all of the respondent's family members. Responses collected as such were submitted to within-subjects MANOVA and found to be highly statistically significant for all subscales (all *p*’s<.001). For the sake of brevity, herein we report only follow-up *t*-tests comparing responses to items as referring to Self, Mother, and Father as was conducted in the prior study. Correlations between item responses to Self, Mother, and Father were also evaluated for significance as was similarly undertaken in the prior study. Tests of convergent validity were conducted by regressing CARTS ratings on responses to the CTQ, CTQ-S, and JVQ-M, whereas tests of concurrent predictive validity for DSM-5 PTSD symptoms were conducted by correlating responses to the CARTS with responses to the PCL-5.

Paired *t*-tests and correlations further examined sibling ratings by way of comparison with parent ratings. In order to be included in these analyses, participants must have included at least one brother or one sister in their family membership. Independent analyses were conducted based on: 1) sibling sex, and 2) whether the sibling was reported to be younger or older than the respondent, yielding four dependent measures (i.e., younger/older brother/sister). For calculations of reliability, as well as paired mean comparisons, in the event that a participant rated multiple siblings within any of the four categories (e.g., more than one younger brother), the first entered sibling of that category was examined. It is important to note that examination of sibling ratings was undertaken independently of the genetic relatedness of siblings to the respondent.

Finally, in order to compare the frequency of occurrences of childhood emotional, physical, and sexual abuse as perpetrated by persons of nuclear familial (e.g., parents, siblings) vs. extra-familial (e.g., aunts, uncles, grandparents) vs. non-familial relationship status (e.g., teachers, coaches, health care professionals, religious persons), we counted separately whether any item from the CARTS Emotional, Physical, and Sexual Abuse subscales was endorsed as referring to each type of relational status, and compared whether the frequency counts differed by relationship status with the Cochran *q*-statistic relative to the chi-square distribution.

## Results

### Internal consistency of CARTS subscales

The obtained Cronbach's alpha coefficients for the CARTS subscales, examined as specific to ratings for 1) “Not Applicable” overall, 2) Self, 3) Mother, and 4) Father, are reported in Table 2. Considering the small number of items per subscale, reliability was determined to be acceptable for most CARTS subscales across different rating types. The internal consistency of the negative affective traits items was lower, particularly in reference to Self, Mother, and Father ratings. Referring to physically abusive items, internal consistency of mother and self-ratings was generally lower compared with that of father ratings. Finally, pertaining to the two items assessing the witnessing of threatened or actual violence directed toward siblings, reliability was generally found to be lower, primarily due to a low correlation between witnessing violence to both a rated brother and a sister. Table 3 reports on the internal consistency statistics for all sibling-rated scales. Cronbach's alpha was good to excellent for most subscales, with similarly lower reliability evidenced for negative affective trait items and physical abuse items.

**Table 2:** Descriptive statistics, paired mean comparisons, and paired correlations across all MTurk participants (*n*=1,782) for not applicable, self, biological mother, and biological father ratings

	Not applicable (a)			Self (b)			Mother (c)			Father (d)			Correlations		
	
Scale (# of items)	*α*	*M*	SD	*α*	*M*	SD	*α*	*M*	SD	*α*	*M*	SD	*r*_bc_	*r*_bd_	*r*_cd_
Positive (13)	.87	0.60	1.71	.90	1.88	3.03	.93	9.27**	4.18	.94	7.85	4.63	.15**	.20**	.43**
Proximity (4)	.86	0.46	1.07				.87	2.34**	1.65	.86	1.38	1.57			.30**
Helps me (4)	.87	0.44	1.05				.89	2.40**	1.69	.89	1.62	1.67			.35**
N-affect (3)	.80	0.76	1.10	.69	0.53	0.87	.66	1.08**	1.10	.63	0.78	0.95	.09**	.08**	.19**
Pos. affect (1)	–	0.09	0.29	–	0.26	0.44	–	0.42**	0.49	–	0.37	0.48	.28**	.29**	.43**
N-feelings from	.89	1.10	1.52				.88	1.25	1.59	.89	1.58**	1.68			.21**
E-Ab to self (2)	.85	0.67	0.88				.86	0.42	0.77	.87	0.46	0.80			.14**
E-Ab to others (2)	.88	0.89	0.93	.69	0.06	0.30	.90	0.36	0.73	.88	0.50**	0.82	.16**	.10**	.22**
N-beliefs from (5)	.90	2.19	2.08				.91	0.91	1.63	.89	1.06**	1.68			.22**
N-beliefs to (5)	.91	2.52	2.13				.90	0.52	1.29	.92	0.93**	1.69			.17**
P-Ab to self (2)	.73	1.56	1.23				.48	0.53	0.90	.64	0.69**	1.03			.28**
P-Ab to others (2)	.81	1.18	0.89	.85	0.04	0.26	.60	0.25	0.55	.77	0.43**	0.73	.11**	.06**	.22**
Wit-V by mom (1)	–	0.71	0.45				–	–	–	–	0.10	0.30			–
Wit-V by dad (1)	–	0.63	0.48				–	0.22	0.42	–	–	–			–
Wit-V by sibs. (2)	.50	1.75	0.54				.12	0.05	0.24	.31	0.04	0.23			.34**
Wit-V to mom (1)	–	0.64	0.48				–	–	–	–	0.24	0.43			–
Wit-V to dad (1)	–	0.79	0.41				–	0.11	0.32	–	–	–			–
Wit-V to sibs. (2)	.59	1.56	0.70				.54	0.14	0.43	.56	0.22**	0.52			.31**
Possible abuse (3)	.87	2.22	1.17				.76	0.15	0.53	.85	0.23**	0.69			.22**
Sexual abuse (6)	.96	5.15	1.92				.93	0.04	0.41	.97	0.21**	1.01			.10**

*p<.05

** *p*<.01. Note that significance level was not corrected for number of comparisons. Ab=abuse; N=negative; P=physical; Pos.=positive; V=violence; Wit=witness.

**Table 3:** Descriptive statistics for brothers and sisters, and paired comparisons with mother and father ratings, across all MTurk participants (*n*=1,364)

	Younger brother			Older brother			Younger sister			Older sister		
	
Scale (# of items)	*α*	*M*	SD	*α*	*M*	SD	*α*	*M*	SD	*α*	*M*	SD
Positive (13)	.88	6.26^a^^c^	3.76	.91	5.74^a^^c^	4.10	.91	6.49^a^^c^	4.06	.93	6.46^a^^c^	4.52
Proximity (4)	.82	0.60^a^^c^	1.15	.85	0.76^a^^c^	1.30	.86	0.73^a^^c^	1.30	.89	1.15^a^	1.56
Helps me (4)	.89	0.74^a^^c^	1.35	.88	0.93^a^^c^	1.45	.88	0.85^a^^c^	1.41	.90	1.30^a^	1.63
N-affect (3)	.49	0.41^a^^c^	0.70	.65	0.45^a^^c^	0.81	.61	0.49^a^^c^	0.83	.69	0.64^a^	0.96
Pos affect (1)	–	0.50^b^^d^	0.50	–	0.44^d^	0.50	–	0.54^b^^d^	0.50	–	0.48^d^	0.50
N-feelings from (4)	.76	0.65^a^^c^	1.11	.86	1.05^c^	1.46	.77	0.73^a^^c^	1.16	.82	0.95^c^	1.35
E-Ab to self (2)	.84	0.44	0.76	.85	0.65^b^^d^	0.87	.87	0.42	0.76	.84	0.60^b^^d^	0.85
E-Ab to others (2)	.85	0.16^a^^c^	0.51	.88	0.31^c^	0.68	.82	0.22^a^^c^	0.57	.82	0.28^c^	0.64
N-beliefs from (5)	.85	0.56^a^^c^	1.26	.87	1.00	1.61	.88	0.71^a^^c^	1.42	.90	1.22^b^	1.82
N-beliefs to (5)	.83	0.36^a^^c^	1.00	.92	0.66^b^	1.47	.86	0.38^a^^c^	1.07	.88	0.56^b^^c^	1.29
P-Ab to self (2)	.80	0.50^c^	0.94	.81	0.65^b^	1.05	.74	0.36^a^^c^	0.78	.82	0.38^c^	0.84
P-Ab to others (2)	.81	0.21^a^^c^	0.56	.94	0.30^b^^c^	0.70	.76	0.17^a^^c^	0.50	.85	0.18^c^	0.54
Possible abuse (3)	.71	0.04^a^^c^	0.31	.88	0.27^b^	0.78	.75	0.04^a^^c^	0.27	.88	0.14	0.57
Sexual abuse (6)	.95	0.04^c^	0.41	.94	0.27^b^	1.10	.80	0.02^c^	0.27	.93	0.11	0.71

a Significantly lower compared to mean score of mother, *p*<.05

b Significantly higher compared to mean score of mother, *p*<.05

c Significantly lower compared to mean score of father, *p*<.05

d Significantly higher compared to mean score of father. Significance level not corrected for number of comparisons.

### Paired comparisons of CARTS subscales across mother, father, and sibling ratings

Table 2 also displays the results of paired comparisons between biological mother and father ratings for all CARTS subscales. Mothers were rated as more Positive, Helping, and as a source of Proximity Seeking than were fathers. Mothers were also rated as having greater overall Negative Affect and Positive Affect. In comparison, fathers were rated as more emotionally abusive to other family members, more physically abusive to the respondent as well as toward other family members, and more sexually abusive toward the respondent. In addition, fathers were rated as more often the source of self-referential negative feelings and beliefs, and more often the target of negative beliefs. However, mothers and fathers were rated similarly with respect to their emotionally abusive behavior toward the respondent.

Table 3 displays the results of paired comparisons between sibling and parental ratings for all CARTS subscales. Paired samples *t*-tests demonstrated that siblings were rated as less Positive, less a source of Proximity Seeking, less Helping, and less Negative in emotional expression when compared with biological mothers and fathers. Younger siblings, in particular, were rated as demonstrating more Positive Affect when compared with both mothers and fathers, while older siblings were rated as demonstrating more Positive Affect only when compared with fathers. Younger siblings were always rated as either less or equally emotionally, physically, and sexually abusive when compared with mothers and fathers, whereas older siblings were always rated as either more or equivalently abusive when compared with mothers and fathers, a trend that was particularly strong in the case of older brothers.

Novel to the current study involved analyses of items referring to the witnessing of domestic violence. Fathers were rated as being more threatening and abusive toward the respondent's mother than vice versa, and were also rated as being more threatening and violent toward the respondents’ siblings. It was statistically infrequent for respondents to endorse witnessing their siblings being violent toward their mother or father, and ratings of witnessing violence did not differ significantly between violence directed by siblings toward mother vs. father.

### Correlations between CARTS subscales across self, mother, father, and sibling ratings

Correlations between CARTS subscales rated in terms of their descriptiveness for self vs. mother vs. father are reported in the final section of Table 2. For all CARTS subscales, mother and father ratings were significantly positively correlated in the small to moderate range. Parent ratings referring to positively framed items, as well as to items assessing negative and positive affect, emotional abuse toward other family members, and physical abuse toward other family members, were also significantly correlated with self-ratings for the same items.

Regarding items assessing witnessing of domestic violence it was found that, when a respondent's father was rated as being abusive toward mother, it was also likely that his or her mother was rated as being abusive toward father (i.e., intimate partner violence was frequently reported to be bidirectional, *r*=.27). Furthermore, there was a positive correlation between ratings of mothers and fathers (*r*=.32) being violent toward respondents’ siblings. Finally, although threats or violence by siblings toward parents were infrequently reported, a positive correlation (*r*=.34) between such ratings indicates that, in families where siblings were violent toward parents, this was usually directed toward both parents rather than only toward either single parent alone.

### Comparison of frequency of occurrence of CARTS emotional, physical, and sexual abuse as perpetrated by persons of nuclear familial vs. extra-familial vs. non-familial relationship status

Table 4 displays the frequency of emotional, physical, and sexual abuse as committed by persons of various relationship status. Consistent with predictions, perpetration of all forms of abuse by nuclear family members occurred far more frequently than did abuse perpetrated by extra-familial members, which in turn occurred far more frequently than abuse perpetrated by non-familial members. All Cochran's *q-*statistics were highly statistically significant in this regard: emotional abuse, *q*(2)=1645.34, *p*<.001; physical abuse, *q*(2)=1576.20, *p*<.001; and sexual abuse, *q*(2)=146.43, *p*<.001.

**Table 4:** Frequency of emotional, physical, and sexual abuse across various nuclear-, extra-, and non-familial relationships

		Emotional abuse		Physical abuse		Sexual abuse	
		
Relationship type	Total entered	*n*	%	*n*	%	*n*	%
Biological father	1,782	412	23.1	530	29.7	69	3.9
Biological mother	1,782	383	21.5	445	25.0	20	1.1
Brother	1,458	369	20.7	311	17.5	57	3.2
Sister	1,178	267	15.0	175	9.8	19	1.1
Nuclear family aggregate	n/a	1,077	60.4	969	54.4	160	9.0
Grandfather	423	14	0.8	14	0.8	9	0.5
Grandmother	646	53	3.0	38	2.1	4	0.2
Step-father	201	68	3.8	53	3.0	37	2.1
Step-mother	111	38	2.1	21	1.2	2	0.1
Mother's partner	43	21	1.2	12	0.7	7	0.4
Father's partner	13	3	0.2	2	0.1	0	0
Uncle	437	32	1.8	15	0.8	28	1.6
Aunt	500	32	1.8	12	0.7	12	0.7
Extra-familial aggregate	n/a	213	12.0	145	8.1	85	4.8
Teacher	45	3	0.2	2	0.1	4	0.2
Doctor	9	0	0	0	0	1	0.1
Coach	5	1	0.1	0	0	0	0
Religious person	23	0	0	0	0	2	0.1
Non-familial aggregate	n/a	4	0.2	2	0.1	6	0.3

Frequency was calculated as a total of whether a person endorsed experiencing any amount of emotional, physical, or sexual abuse (i.e., a score of 1 on any item in the scale). If multiple persons were entered for a particular category (e.g., sister), any abuse committed by any entered person was tallied. Percentages were calculated as the *Total*/1,782.

### Convergent validity of the CARTS with other childhood trauma measures

Table 5 reports the results of multiple regressions evaluating the convergent validity of the CARTS with the JVQ-M subscale, the CTQ subscales, and the CTQ-S items. CARTS ratings accounted for 49% of the variance in the JVQ-M. CARTS ratings accounted for 15–20% of the variance across the CTQ-EA, CTQ-PA, and CTQ-SA. Finally, CARTS ratings accounted for 15–21% of the variance across the CTQ-S subscales. Inclusion of CARTS ratings specifically referring to a participants’ mother and father incrementally predicted additional variance in these other childhood trauma measures (CTQ, JVQ, CTQS) above what was accounted for by the number of CARTS items that were regarded as not-applicable across relationship types, except in the case of CTQ-measured sexual abuse histories.

**Table 5:** Multiple regression analyses of CARTS convergent validity with the Childhood Trauma Questionnaire, the Juvenile Victimization Questionnaire, and the childhood trauma screen

		Step 1: Non-applicable ratings	Step 2: Mother & father ratings		Non-applicable ratings	Mother ratings	Father ratings
				
Dependent measure	Sub-sample	*R* ^2^	Δ*R* ^2^	(CARTS predictor)	*b* (*SE*)	*b* (*SE*)	*b* (*SE*)
CTQ-EA	1	.10**	.05**	EA	−.20 (.32)**	.18 (.37)**	.22 (.34)**
CTQ-PA	1	.08**	.02**	PA	−.19 (.20)**	−.003 (.22)	.17 (.21)**
CTQ-SA	1	.19**	.01	SA	−.40 (.13)**	−.02 (.46)	.09 (.24)*
JVQ-M	2	0.45**	.09**	EA	−.20 (.40)**	.23 (.52)**	.18 (.48)**
				PA	−.26 (.33)**	.10 (.50)	−.02 (.41)
				SA	−.18 (.19)**	−.14 (.70)**	−.002 (.31)
CTQ-S-EA	3	.002	.15**	EA	−.11 (.08)	.44 (.09)**	.55 (.09)**
CTQ-S PA	3	.003	.21**	PA	−.04 (.05)	.23 (.07)**	.33 (.05)**
CTQ-S SA	3	.001	.19**	SA	−.03 (.03)	.14 (.16)**	.40 (.06)**

CTQ=Childhood Trauma Questionnaire; CTQ-S=Childhood trauma screen; JVQ-M=Juvenile victimization questionnaire-maltreatment scale; EA=Emotional abuse; PA=Physical abuse; SA=Sexual abuse. Prediction equations of CTQ scales correspond to CARTS scale for the specific form of abuse; for example, CTQ-EA predicted with CARTS emotional abuse to self-scale for a given family member.

** *p*<.01

* *p*<.05.

### Concurrent predictive validity for symptoms of posttraumatic stress

Table 6 reports the results of correlations between the PCL-5 and those CARTS subscales scored for various relationship types. Statistically significant though generally small correlations were observed between most CARTS subscales referring to self, biological parents, and younger sisters. Correlations between PCL-5 and abuse perpetrated by grandmothers, step-fathers, mother's partners, and uncles were also observed. By contrast, associations with abuse perpetrated by persons of non-familial status were non-significant.

**Table 6:** Correlations between CARTS subscales and PCL-5 total scores separately for each assessed relationship

	Total completed	Positive	Proximity seeking	Helping	Neg. affect	Pos. affect	Neg. feel. to	Emot. abuse self	Emot. abuse others	Relate. beliefs from	Relate. beliefs to	Phys. abuse self	Phys. abuse others	Possible abuse	Sexual abuse
Not-applicable	1,685	.03	.01	.03	−.16**	.02	−.17**	−.13**	−.13**	−.17**	−.15**	−.11**	−.12**	−.15**	−.14**
Self	1,685	−.11**			.17**	−.13**			.05				.05		
Mother	1,685	−.20**	−.16**	−.18**	.15**	−.14**	.16**	.13**	.11**	.20**	.19**	.12**	.10**	.09**	.06
Father	1,685	−.16**	−.10**	−.12**	.17**	−.11**	.14**	.13**	.11**	.19**	.13**	.13**	.12**	.14**	.14**
Younger brother	417	−.20**	−.05	−.06	.02	−.05	.12*	.05	.07	.11	.05	.01	.04	.07	.08
Older brother	478	−.06	.02	−.01	.08	−.07	.11	.09	.11	.11	.18**	.10	.09	.14**	.03
Younger sister	376	−.10	.01	−.01	.14**	−.20**	.24**	.16**	.22**	.24**	.23**	.22**	.18**	.06	.07
Older sister	395	−.10	−.05	.01	.01	.01	.03	.05	−.01	.07	−.01	.01	.01	.10	.18**
Nuclear family aggregate	1,627							.11**				.08**			.13**
Grandfather	303							.03				.06			.06
Grandmother	443							.10**				−.08			.04
Step-father	171							.14*				.20**			.07
Step-mother	99							−.01				.12			−.01
Mother's partner	38							.24				.33**			.08
Father's partner	12							.23				.19			–
Uncle	242							.16**				.12*			.19**
Aunt	268							−.03				.07			.04
Extra-familial aggregate	653							.08*				.09*			.08*
Nonfamilial aggregate	41							−.14				.03			−.01

Total completed column refers to those who entered both the relationship category of the CARTS and the PCL.

* *p*<.05

** *p*<.01. Aggregate variables are summed scores and include the same categories as presented in Table 4.

## Discussion

Childhood abuse is often experienced within the complex social microsystems of families and peer relationships, as well as within the greater ecological environments that encapsulate families (e.g., communities, institutions). However, most current psychometric assessment tools for measuring occurrences of childhood maltreatment fail to take any explicit account of the complex family dynamics and socioecological context within which childhood maltreatment occurs. We therefore undertook the task of further evaluating the CARTS as a novel methodology for assessing the family dynamics and relational socioecology within which childhood maltreatment often occurs. Beyond further establishing the internal reliability of the CARTS, its convergent validity with other standard surveys of childhood trauma history, and its concurrent predictive validity for symptoms of posttraumatic stress, the current study serves to detail how the assessment approach implemented by the CARTS begins to describe some of the relational complexities inherent to many experiences of childhood trauma, neglect, and attachment disturbance.

Replicating previous findings with the CARTS (Frewen et al., 2013), mothers were regarded as more positive, secure, and emotionally available to their children when compared with fathers (Lum & Phares, 2005). In addition, respondents’ mothers were rated as displaying both more negative and positive affect than fathers, suggestive of mothers being generally more emotionally expressive than fathers independent of emotional valence. Also consistent with previous findings, fathers were more often rated than were mothers as being emotionally, physically, and sexually abusive, the source of negative self-referential feelings and beliefs, and the target of negative relational beliefs (Frewen et al., 2013). How mothers and fathers were rated across the CARTS subscales was consistently associated with current symptoms of posttraumatic stress.

Beyond examination of parental figures, the current study is the first to examine sibling ratings on the CARTS. In comparison with parents, siblings were rated as less emotionally available to respondents, and less positive in terms of their respective relationship (Aguilar, O'Brien, August, Aoun, & Hektner, 2001). Overall, younger siblings were also rated as less likely to commit acts of emotional, physical, and sexual abuse toward the respondent and other family members when compared with parents, and were also regarded as less often the source and target of negative self-referential feelings and beliefs as compared with parents. However, as compared with mothers and fathers, older siblings, and in particular older brothers, were rated as being more emotionally and physically abusive toward the respondent directly, consistent with prior work (Aguilar et al., 2001; Button & Gealt, 2010; Menesini, Camodeca, & Nocentini, 2010).

With regard to sex differences, the current study is consistent with prior findings regarding the greater prevalence of intersibling violence between an older male perpetrator and a younger victim, as well as the detrimental impact such violence can have on psychological outcomes (e.g., Eriksen & Jensen, 2006, 2009; Tippett & Wolke, 2014). Our findings are consonant with research suggesting that siblings who feel that they are dissimilar (e.g., one sibling is substantially older) tend to have a less cooperative relationship (i.e., low positivity, proximity seeking, and helping behavior) and higher levels of conflict and abuse (e.g., Graham-Bermann, Cutler, Litzenberger, & Schwartz, 1994; Tippett & Wolke, 2014). Importantly, our findings suggest that such an outcome is most likely when the older sibling is male (Duncan, 1999; Menesini et al., 2010; Tippett & Wolke, 2014; Tucker et al., 2013; Wolke, Tippett, & Dantchev, in press). At the same time, siblings may have been rated lower on items pertaining to emotional and physical abuse of other family members due to participants often reporting small family sizes, where the respondent themselves may be the only potential target of abuse excepting between parents. Interestingly, CARTS ratings referring to younger sisters were also consistently associated with posttraumatic stress. Future research should determine whether abuse perpetrated by a younger sister somehow has a uniquely adverse impact, or whether abuse perpetrated by a younger sister rather acts as a particularly salient marker of dysfunctional family dynamics.

We also reported on an extended CARTS item set assessing the witnessing of interparental and parent-to-child violence in order to investigate additional aspects of family dysfunction (see Appendix). Fathers were rated as more abusive (threatening or committing assault) toward mothers and to the respondents’ siblings than vice versa. Given the retrospective nature of our study, respondents may have been more likely to report witnessing incidences of male-to-female violence, because violence committed by men is typically of greater severity and more likely to result in physical injury (e.g., Tjaden & Thoennes, 2000). However, incidences of intimate partner violence are frequently bi-directional in nature, and prevalence of female-to-male violence has likely been underreported (Archer, 2000; Bates, Graham-Kevan, & Archer, 2014). Findings from the current CARTS research tended to confirm the bi-directionality of intimate partner violence as indicated by correlations between reported witnessing of violence directed between father-to-mother and mother-to-father (Anderson, 2002; McKinney et al., 2009; Renner & Whitney, 2010; Straus, 2008).

New to the current study was the examination of ratings for extra-familial and non-familial family members, and comparison of incidence rates of emotional, physical, and sexual abuse perpetrated by such persons relative to the nuclear family (biological parents and siblings). Obtained results strongly support epidemiological research documenting the greater prevalence of childhood abuse committed at the hands of primary caretakers (e.g., biological parents) and other core family members (i.e., siblings) in comparison with non-family members (e.g., Finkelhor et al., 2014). Moreover, measures of posttraumatic stress were more strongly associated with CARTS ratings referring to adults with probable caregiving roles (biological mother, biological father, step-father, mother or father's partner), in comparison with ratings referring to extra-familial adults (e.g., coaches, doctors, teachers, religious persons).

We acknowledge the limitations of our research. First, our study evidenced a drop-out rate of 17.2%, and those who completed the study were more likely to report a diagnosed psychological problem than those who dropped out; our results may therefore lack generalizability to the general population, and may instead be better descriptive of a population evidencing a greater than average prevalence of mental health problems. Furthermore, MTurk populations are known to differ from the general population on certain demographic characteristics, being generally of younger average age, more educated, and evidencing a lower likelihood of employment. Second, the CARTS is a retrospective self-report survey and therefore is subject to recall bias. Third, although our sample included both men and women of ranging ethnicity, females were overly represented, as were Caucasians and more educated persons, potentially further limiting generalizability. Fourth, although analyses expanded beyond a previous study examining only parental abuse (Frewen et al., 2013) to include analysis of ratings referring to siblings, we analyzed only the first reported brother or sister in each sibling category, which may introduce measurement bias; this problem is not inherent to the CARTS but rather to the analyses conducted herein, the potential impact of which might be evaluated in future studies.

Additional lines of future inquiry for the CARTS include examination of the effect of additional family-level factors such as family resources and parental mental or physical illness, all of which have been shown to influence child mental health and conceivably affect perceptions of parents and siblings (e.g., Armistead, Klein, & Forehand, 1995; Bøe et al., 2014; Manning & Gregoire, 2009). Researchers may also wish to devise shorter versions of the item set used herein, based on item response theory or other item reduction techniques, or apply the CARTS relational response format to the investigation of other validated survey items. Further investigation into the nature of sibling violence could also be pursued. Specifically, the current research did not determine whether identical items nevertheless imply different levels of severity when directed toward different persons, for example, parents vs. siblings. For example, an item such as “This person, slapped, smacked, or hit me” may be considered indicative of highly abusive behavior when applied to a parent, but perhaps considered a relatively normative experience when applied within the context of certain sibling or peer relationships. Although one might expect that the general context typically in place when participants are surveyed about their personal trauma history would tend to encourage a reserved stance toward item interpretation and endorsement, future research should confirm that item endorsements imply their intended interpretations through such methodologies as “think-aloud” experiments.

In summary, evidence from the current study further support the CARTS as a novel survey methodology for assessing the warmth, security, and supportiveness of early attachment relationships, and the complex family and relational dynamics often underlying or surrounding occurrences of childhood trauma. Mental health professionals and researchers using a family or relationally oriented approach to conceptualizing attachment security and the measurement of maltreatment experiences may find administration of the CARTS useful. Further investigation of this assessment approach seems warranted.

## Supplementary Material

Assessing the family dynamics of childhood maltreatment history with the Childhood Attachment and Relational Trauma Screen (CARTS)

## Appendix

**Witnessed violence by mother**

I witnessed (watched or heard) this person being threatened or assaulted by my mother

**Witnessed violence by father**

I witnessed (watched or heard) this person being threatened or assaulted by my father

**Witnessed violence by siblings**

I witnessed (watched or heard) this person being threatened or assaulted by one or more of my brothers

I witnessed (watched or heard) this person being threatened or assaulted by one or more of my sisters

**Witnessed violence towards mother**

This person threatened or assaulted my mother

**Witnessed violence towards father**

This person threatened or assaulted my father

**Witnessed violence towards siblings**

This person threatened or assaulted one or more of my brothers

This person threatened or assaulted one or more of my sisters
