# Supplementary material for: Assessing the family dynamics of childhood maltreatment history with the Childhood Attachment and Relational Trauma Screen (CARTS)
Source: Eur J Psychotraumatol. 2015 Aug 3;6:10.3402/ejpt.v6.27792. doi: 10.3402/ejpt.v6.27792 (PMC4524890; doi:10.3402/ejpt.v6.27792)
Supplement: Assessing the family dynamics of childhood maltreatment history with the Childhood Attachment and Relational Trauma Screen (CARTS) [file EJPT-6-27792-s001.pdf]

Metody pomiaru dynamiki historii dziecięcego maltretowania w rodzinie, dziecięgo przywiązania oraz traumy relacyjnej.

Paul Frewen, Matthew Brown, Jonathan DePierro, Wendy D'Andrea, Allan Schore

Obecne narzedzia pomiaru traumy dziecięcej nie uwzgledniają relacyjno-socjoekologicznych aspektów srodowiska, w którym wychowuje się dziecko. Takie zmienne jak relacja pomiedzy sprawca a ofiarą, emocjonalna dostępność opiekunów, bycie świadkiem przemocy czy własne emocje, myśli i działania towarzyszące maltretowaniu rzadko są brane pod uwagę w takich narzędziach. Aby wypełnić ta lukę badawczą, w pracy tej analizowaliśmy dziecięce maltretowanie przy użyciu narzędzia Childhood Attachment and Relational Trauma Screen (CARTS) na próbie 1782 osób zbadanych przez internet. Ogólne wyniki naszych badań wskazują, że relacja badanych z biologicznymi ojcami była mniej satysfakcjonująca i mniej bezpieczna w porównaniu do relacji z biologicznymi matkami i to własnie biologiczni ojcowie byli najczęściej sprawcami przemocy emocjonalnej, fizycznej oraz seksualnej.
Dodatkowo wykazaliśmy, że emocjonalne, fizyczne oraz seksualne wykorzystanie w dzieciństwie było najczęściej dokonywane przez członków najbliższej rodziny, a nie dalszych krewnych. Wyniki naszych badań wskazują na zasadność wykorzystania narzedzia hildhood Attachment and Relational Trauma Screen (CARTS) do analizy dynamiki maltretowania w dzieciństwie.

Name of translator: Marcin Rzeszutek, University of Finance and Management in Warsaw, Poland

Citation: European Journal of Psychotraumatology 2015, 6: 27792 - http://dx.doi.org/10.3402/ejpt.v6.27792
